# Multimodal Fake-News Recognition Using Ensemble of Deep Learners

**DOI:** 10.3390/e24091242

**Published:** 2022-09-03

**Authors:** Abdulhameed Al Obaid, Hassan Khotanlou, Muharram Mansoorizadeh, Davood Zabihzadeh

**Affiliations:** 1 RIV Lab, Department of Computer Engineering, Bu-Ali Sina University, Hamedan 65178-38695, Iran; 2 Thi Qar Governorate Council, Al Nasiriyah 64001, Iraq; 3 Department of Computer Engineering, Hakim Sabzevari University, Sabzevar 96179-76487, Iran

**Keywords:** fake-news recognition, ensemble of deep learners, attention mechanism, multimodal data

## Abstract

Social networks have drastically changed how people obtain information. News in social networks is accompanied by images and videos and thus receives more attention from readers as opposed to traditional sources. Unfortunately, fake-news publishers often misuse these advantages to spread false information rapidly. Therefore, the early detection of fake news is crucial. The best way to address this issue is to design an automatic detector based on fake-news content. Thus far, many fake-news recognition systems, including both traditional machine learning and deep learning models, have been proposed. Given that manual feature-extraction methods are very time-consuming, deep learning methods are the preferred tools. This study aimed to enhance the performance of existing approaches by utilizing an ensemble of deep learners based on attention mechanisms. To a great extent, the success of an ensemble model depends on the variety of its learners. To this end, we propose a novel loss function that enforces each learner to attend to different parts of news content on the one hand and obtain good classification accuracy on the other hand. Also, the learners are built on a common deep-feature extractor and only differ in their attention modules. As a result, the number of parameters is reduced efficiently and the overfitting problem is addressed. We conducted several experiments on some widely used fake-news detection datasets. The results confirm that the proposed method consistently surpasses the existing peer methods.

## 1. Introduction

Obtaining news from social media has become increasingly prevalent. Nowadays, more people acquire news from social media. Social networks benefit from providing multimedia information for news, with low cost, ease of access, and rapid dissemination. These advantages increasingly attract many people to consume news through them. Unfortunately, these features often are misused by fake-post publishers to spread the news rapidly. The rapid dissemination of fake news may cause negative impacts on society or can even alter the outcomes of an important public event. Thus, early fake-news detection on social media has recently become an active field and has attracted widespread attention.

Fake news is intentionally and verifiably false and could mislead its readerships [1]. A news article contains two major components: publisher and content. The publisher includes a set of features that identify the author, such as name, age, domain, etc. The content consists of a set of attributes that represent the news article, such as title, body copy, images, videos, etc.

For a given post a, the task of fake-news recognition is to predict whether the article is fake or not. This task is often modeled as a binary classification. Although other sources, such as users’ comments about the article and reposts, are indeed helpful, this information in the early stages is often incomplete and noisy. Thus, this work is focused on detecting fake news based on the content.

Thus far, many fake-news recognition systems, including traditional machine learning and deep learning models, have been proposed. Traditional methods first manually extract features from news and then classify them by exploiting these features. In contrast, deep learning models can automatically extract useful features from text or images in the news. Since manual feature-extraction methods are very time-consuming, deep learning methods are preferred over traditional ones.

Ensemble is a popular technique to train multiple models for achieving a composite model, which outperforms individual learners. To a great extent, the success of an ensemble model depends on both the accuracy of each learner and the diversity among them. Ideally, each learner should have high accuracy and must have a low correlation with others.

Existing ensemble fake-news methods [2,3,4,5,6,7,8,9,10] often trained multiple deep or shallow models independently and then combined the outcomes of learners via ensemble mechanisms, such as voting. Thus, these models involve many trainable parameters and a costly training process. Also, they suffer from the scalability aspect and are vulnerable to the overfitting problem.

**Figure 1:**
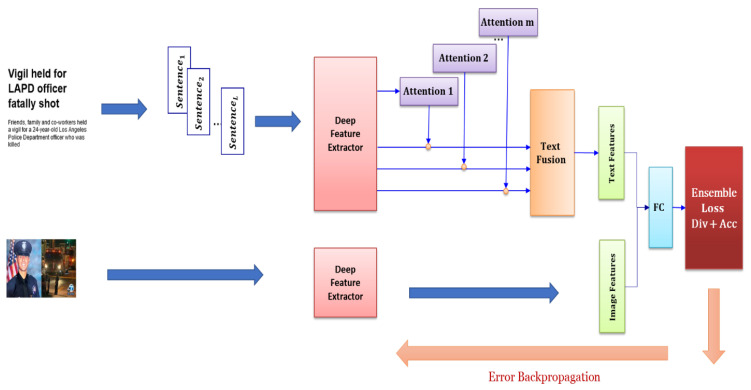
Overall structure of the proposed ensemble of deep learners. The learners are built on top of a shared feature extractor and have a different attention layer. The model was trained using the proposed ensemble loss that enforces diversity among learners and encourages them to have high accuracy.

To address these challenges, we propose a novel fake-news detector that utilizes an ensemble of deep learners and attention mechanisms. Our learners are built on top of a *shared* deep-feature extractor and differ in their *attention* modules. Sharing parameters effectively reduces training time, memory requirements, and the complexity of the proposed model. Also, our model is less prone to the overfitting problem. Figure 1 shows the proposed architecture. The proposed text feature extractor is similar to the hierarchical attention network (HAN) [11], with the difference being that we obtained sentence representation from the pretrained XLNet [12], which is much faster than word embedding, followed by bidirectional GRU and attention in HAN. Moreover, we have multiple attention modules to encode the input document. Here, the proposed loss function forces each module to attend to the different parts of the document on one hand and attain good classification accuracy on the other hand.

The proposed ensemble model is trained in an end-to-end paradigm using the standard of backpropagation (BP).

In summary, the contributions of this paper are as follows:

1. We propose an ensemble of deep learners built on a shared feature extractor to identify fake news. Our model has fewer parameters and training time compared with existing ensemble models. As a result, it is less prone to the overfitting problem.
2. We develop a novel loss function that enforces each learner to focus on a different aspect of the input news using an attention mechanism. This encourages each model to have high performance. The model is trained in an end-to-end learning paradigm using the standard BP.

The remainder of the paper is organized as follows. Section 2 reviews related fake-news detection methods with a focus on multimodal content-based approaches. Section 3 presents the proposed ensemble model and its implementation details. Section 4 reports the experimental results and comparisons with peer state-of-the-art methods. Finally, Section 5 concludes with remarks along with recommendations for future work.

## 2. Background

A major challenge in a fake-news detection task is the classification of news using available features. Features can be derived from many sources, such as body copy, attached images or videos, user profiles, social context, users’ comments, or reposts. The early discovery of fake news mostly relies on its content. A list of possible features of content include:

1. Author or publisher of the news.
2. Title: summary text intended to draw readers’ attention to the main topic of the news.
3. Body copy: the main body of news that describes the details of the news. News stories are usually a big claim that shows the direction of the publisher.
4. Images/videos: Part of a news article that provides visual cues for the story.

Here, we review related work from three categories: (1) single modal, (2) multimodal (text and image), and (3) ensemble approaches.

### 2.1. Single Modality-Based Methods

Textual attributes refer to statistical or semantic features extracted from the content of the text and have been studied in fake-news discovery literature [1,13,14,15]. For example, Reis et al. [16] used several types of features, such as language (syntax), lexical, psycholinguistic, semantic, and news sources. Afterward, they applied several machine learning methods, such as kNN, naïve Bayes, SVM, and random forest to the extracted features and compared the results.

Unfortunately, linguistic patterns are not yet well understood, because they depend on specific events and domain-related knowledge [17]. Therefore, it is difficult to design manual features from the text for the traditional machine learning models. To overcome this limitation, deep learning models are utilized to automatically extract features and identify fake news simultaneously.

Inspired by the pioneering work in [11], De Sarkar et al. [18] presented a deep learning model for fake-news detection with two major components: the *S* and *D* modules. The *S* module generates a sentence embedding for each sentence in the source news. The *D* module takes sentence embedding as input and creates document embedding using an attention mechanism.

HDSF (hierarchical discourse-level structure for fake-news detection) [19] incorporated a hierarchical discourse-level structure of fake and real news articles. This method is developed based on the dependence parsing of the document at the sentence level using bidirectional LSTM.

FakeBert [20] proposed a BERT (bidirectional encoder representations from transformers)-based [21] encoder that helped it to obtain a deeper sense of news context. Here, the BERT encoder has been followed by several parallel 1-D convolutional blocks with different kernel lengths. The outputs of the blocks were concatenated and passed to a dense classifier. The reported results show that the BERT outperforms unidirectional embeddings, such as Glove [22].

### 2.2. Multimodal Methods

Visual cues contain valuable information to detect fake news [1,23]. While few studies have focused on validating the multimedia content of the news, some research has considered these features [23,24,25,26].

TI-CNN (text and image information-based CNN) [27] combined the explicit and latent features of text and image information into a unified feature space and then used the learned features to identify fake news.

EANN (event adversarial neural networks for multimodal fake-news detection) [28] is a multimodal approach aimed at learning an event-invariant representation using domain-adaptation techniques. In this way, it removes tight dependence on the specific events in the collected dataset and achieves better generalization ability on the unseen events.

As these studies indicate the importance of visual features, we consider the visual content in our model.

### 2.3. Ensemble Methods

Several ensemble models for fake-news recognition have been presented in the literature to enhance the accuracy of the task. Rot et al. [6] developed two models based on CNN and bidirectional LSTM to extract features from news and then passed the obtained representations to an MLP model for the final classification.

Huang et al. [5] set up an ensemble model using four deep models, namely, embedding LSTM, depth LSTM, LIWC CNN, and N-gram. It utilized a metaheuristic method named “self-adaptive harmony search” (SAHS) to find the optimized weights of each model [10].

Hakak et al. first extracted important features from fake-news datasets and then passed the features to three popular traditional learning algorithms, namely, decision tree, random forest, and extra tree classifier. Similarly, Mahabub et al. [4] investigated 11 traditional machine learning algorithms, such as kNN, SVM, and random forest, for detection. Then, they selected the three best models using cross-validation and combined the results using the voting mechanism. Hansrajh et al. [9] trained logistic regression, linear discriminant analysis (LDA) classifier, SVM, stochastic gradient descent, and ridge regression on the LIAR dataset [29]. Then, it employed “blending,” a variant of the stacking mechanism, to fuse the predictions of base models. Iftikhar et al. [10] added several linguistic features obtained via the LIWC2015 tool from the text content to the evaluated datasets. It then examined the performance of several base models and ensemble mechanisms, including bagging, boosting (Adaboost and XGBoost), voting, and random forest on four datasets.

Aslam et al. [3] proposed an ensemble model to identify fake news on the LIAR dataset. It used the bidirectional LSTM-GRU model for the textual content of the input post and a MLP model for other features. The outputs of the models were concatenated and formed the embedding representation. The representation was then forwarded to a single fully connected layer with one output neuron [9,30].

Meel et al. [8] proposed a multimodal ensemble fake-news detection model that utilized a hierarchical attention network (HAN) for text feature extraction and image captioning to extract visual features. It also implemented headline matching with text content, noise variance inconsistency, and error level analysis algorithms. The algorithms were trained independently and combined using the max voting mechanism. Compared to the proposed method, we experimentally showed that our ensemble-based text feature extractor outperformed HAN on both evaluated datasets on a variety of metrics (refer to Section 3.4). Also, while the authors showed that the combined model achieves promising results, the model demands costly training and evaluation processes.

Das et al. [7] used a variety of pretrained network models to extract features from news text content. Each model is followed by an output layer that produces probabilities for real and fake classes. Afterwards, it used soft voting and hard voting to combine the predictions. Additionally, it presented an heuristic post-processing approach that boosted the F1 score of the ensemble from 98.31% to 98.31% on the COVID-19 fake-news dataset from the CONSTRAINT COVID19 competition, thereby achieving state-of-the-art performance on this dataset. However, the current results indicate that several teams obtained an accuracy of 100% and surpassed it.

As observed, existing methods often train multiple independent models and then combine the results using ensemble mechanisms. In the case of using deep networks as base learners, this strategy requires many parameters and a costly training process. These models are also prone to the overfitting problem.

## 3. Materials and the Proposed Method

The proposed method, called “diverse ensemble of fake-news detectors” (DEFD) aims to detect fake news based on its content. To understand the meaning of news, we need to process the sentences that form it. Considering that different sections of a post do not equally contribute to identifying its fakeness, we utilize an attention mechanism to automatically learn their importance weights.

The proposed model consists of a consensus of learners that have a common structure to simplify the model and prevent overfitting. The difference among these learners only lies in their attention modules. Given that the success of an ensemble model depends on how different the learners are, we aim to have each learner consider various aspects of the post. Thus, we try to make them as different as possible in the proposed loss function.

**Figure 2:**
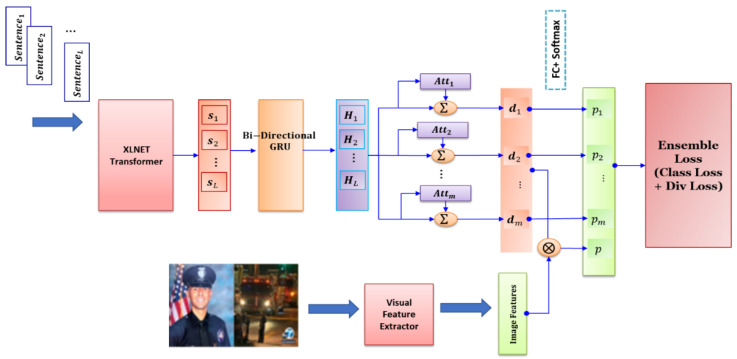
Architecture of the proposed ensemble of deep learners. Each learner has a specific attention module and a Softmax layer. The proposed ensemble loss receives predictions. It enforces diverse predictions and encourages learners to have high classification accuracy.

Figure 2 illustrates the architecture of the proposed model, in which two parallel components are utilized to extract features from the image and text of given news. Then, the extracted features are merged and categorized. In the following, different parts of the proposed model are discussed in more detail.

### 3.1. Text Feature Extraction

Our text feature extractor has three components: sentence encoder, post encoder, and attention modules.

#### 3.1.1. Sentence Encoder

Words are first converted to a fixed-length vector using a predefined word embedding such as BERT [21], word2vec [31], Glove [22], or FastTex [32].

A sentence can be represented as a sequence of words. Suppose $u_{ij}$ is the jth word of ith  sentence, obtained from a word embedding. Then, a sentence can be denoted by $u_{i1},\;u_{i2},\ldots ,u_{il_{w}}$ where $l_{w}\;$ is the number of words. The sentence encoder should map this sequence to a vector with a fixed-length $d_{w}$. In other words, it can be modeled by a function f, such that $\left(s_{i}=f\left(u_{i1},\;u_{i2},\ldots ,u_{il_{w}}\right)\right)$ where the output $s_{i}$ denotes the embedding of the ith sentence. 

A sentence encoder can be implemented by available deep learning models that work on text data, such as Temporal CNN, GRU, or LSTM, or state-of-the-art transformer models, such as BERT and XLNET [12].

#### 3.1.2. Post Encoder

Each post contains a sequence of sentences. The input of the model is a post. We apply the sentence encoder to each sentence i in the input post to attain its vectorized representation $s_{i}$. The representations of sentences are further processed by a bidirectional GRU to obtain annotations ($H_{i}$) for each sentence $s_{i}$ in the post. Then, we apply attention layers as discussed in the next subsection on these annotations. Subsequently, each attention layer j obtains a representation $d^{\left(j\right)},\;j=1,2,\ldots ,m$ for the given post. Also, we consider an additional representation $d=d^{\left(m+1\right)}=concat\left(d^{\left(j\right)},\;j=1,2,\ldots ,m\right)$ that provides the whole view of learners from the input document.

#### 3.1.3. Attention Module

**Figure 3:**
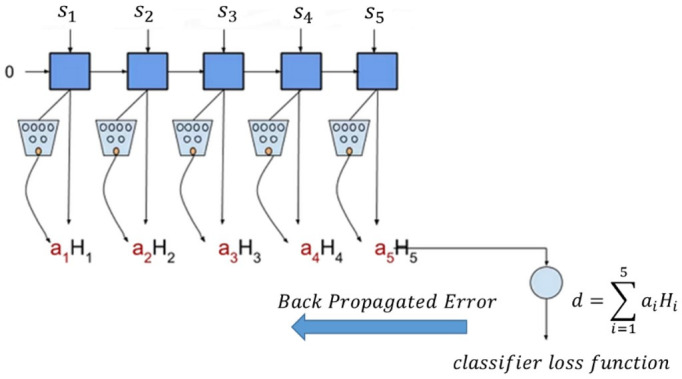
Simple representation of an attention mechanism in a post with five sentences.

The attention module aims to assign larger weights to the more important parts of given news. As an illustrative example, suppose a post contains five sentences. These sentences are processed by a deep network, such as, a bidirectional LSTM, and a state variable is generated at each step (these variables are represented by H in Figure 3). $H_{1}$ deals mostly with the first few sentences of the text, such as $s_{1}$ and $s_{2}$, whereas $H_{5}$ focuses on the last sentences. The attention module, which is usually implemented by a simple two-layer neural network, has the task of attaining the attention weights,$\;a_{i},\;i=1,2,\ldots ,5$. The post embedding d is then formed by weighted averaging of the states: $d=\overset{5\;}{\underset{i=1}{\sum }}a_{i}H_{i}.$

The attention mechanism is based on a similarity function and a context vector, such as $q\in {\mathbb{R}}^{e}$ that we aim at attending. First, the alignment scores $g\left(H_{i},q\right)$ (for each $H_{i}\in {\mathbb{R}}^{e}$ $i=1,2,\ldots ,l_{s}$ and q) are computed. The function $g\left(H_{i},q\right)$ measures the similarity between $H_{i}$ and q or the attention of $H_{i}$ to q. Second, the attention weights are normalized by applying the *Softmax* function that transforms the similarity scores ${\left[g\left(H_{i},q\right)\right]}_{i=1}^{l_{s}}$ to a probability (or normalized) vector ${\left[a_{i}\right]}_{i=1}^{l_{s}}$. Many different attention mechanisms are proposed in the literature. They mostly differ in the way the alignment score function g is modeled. Below, we review some important ones [33].

- **Multiplicative Attention:**

Multiplicative attention uses inner product similarity to compute $g\left(H_{i},q\right)$ as follows:

(1) $$g\left(H_{i},q\right)=\langle W^{\left(1\right)}H_{i},W^{\left(2\right)}q\rangle .$$

The weight matrices $W^{\left(1\right)}$ and $W^{\left(2\right)}$ are learned using BP according to the objective function of the task.

- **Modified Multiplicative Attention**

This method [11] first applies a linear transformation followed by a tanh activation on each state $H_{i}$ in the input post. Then, it computes the inner product of the result with the context vector q.

(2) $$g\left(H_{i},q\right)=\langle h_{i},q\rangle ,\;\;\;\mathrm{where}\;h_{i}=\tanh\left(W^{\left(1\right)}H_{i}+b^{\left(1\right)}\right).$$

- **Additive Attention**

Additive attention computes the similarity score between $H_{i}$ and q as:

(3) $$g\left(H_{i},q\right)=w^{T}\sigma \left(W^{\left(1\right)}H_{i}+W^{\left(2\right)}q\right).$$

where $w\in {\mathbb{R}}^{e}$ is a weight vector and σ is an activation function, such as tanh. Additive attention often outperforms the multiplicative form. However, it requires more memory and computational cost.

- **Multidimensional Attention**

Multidimensional attention computes multiple scores, one for each feature in $H_{i}$ by replacing the weight vector w in the additive form with a matrix $W\in {\mathbb{R}}^{e\times e}$:

(4) $$g\left(H_{i},q\right)=W^{T}\sigma \left(W^{\left(1\right)}H_{i}+W^{\left(2\right)}q\right).$$

Let ${\left[g\left(H_{i},q\right)\right]}_{j=1}^{e}$ be the scores (components) obtained from the multidimensional attention. Here, the $k{\mathrm{th}}^{}$ dimension of $g\left(H_{i},q\right)$ means the attention of the kth feature of $H_{i}$ to q. Hence, each feature in $H_{i}$ has a score weight. Now, the same procedure is applied to each feature k. First, we normalize the attention weights of feature k, $\left\{{\left[g\left(H_{i},q\right)\right]}_{k}\;i=1,2,3,\ldots ,\;l_{s}\right\}$), using the Softmax function:

(5) $$a_{ik}={\left[\mathrm{softmax}\left({\left[g\left(H_{i},q\right)\right]}_{k}\right)\right]}_{i}=\frac{\exp\left({\left[g\left(H_{i},q\right)\right]}_{k}\right)}{\sum_{j=1}^{l_{s}}\exp\left({\left[g\left(H_{j},q\right)\right]}_{k}\right)}.$$

Then, we form the $k{\mathrm{th}}^{}$ feature of output d as:

(6) $$d_{k}=\;\overset{l_{s}}{\underset{i=1}{\sum }}a_{ik}H_{ik},\;\;i=1,2,\ldots ,m$$

In this work, we adopt the *modified multiplicative attention* to implement the attention mechanism. The main question is: To which feature of vector  q should we attend? It shows a reference representation that a sentence annotation ($H_{i}$) should be similar to it to attain a higher representation score. For some tasks, such as visual question answering, the query vector q is given as input. However, it is not determined in our task. Thus, we define the context vector q as a learnable parameter vector in our model to be learned jointly with other parameters according to the loss function.

### 3.2. Image Feature Extraction

In addition to text, images can also play an important role in determining whether a given post is fake or not. Therefore, we consider visual information in the proposed model. Feature extraction from images can be done by a pretrained CNN. Also, one can extract objects from the input image and use an attention mechanism to force the learner to focus on specific objects in the image. Here, we simply use a pretrained VGG19 [34] to mine features from the given image. The features are then processed by several fully connected (FC) layers, each followed by an activation function. The implementation details are provided in Section 4.3. Finally, as seen in Figure 2, the text and image features are concatenated and then passed to the loss layer.

### 3.3. Loss Function

**Figure 4:**
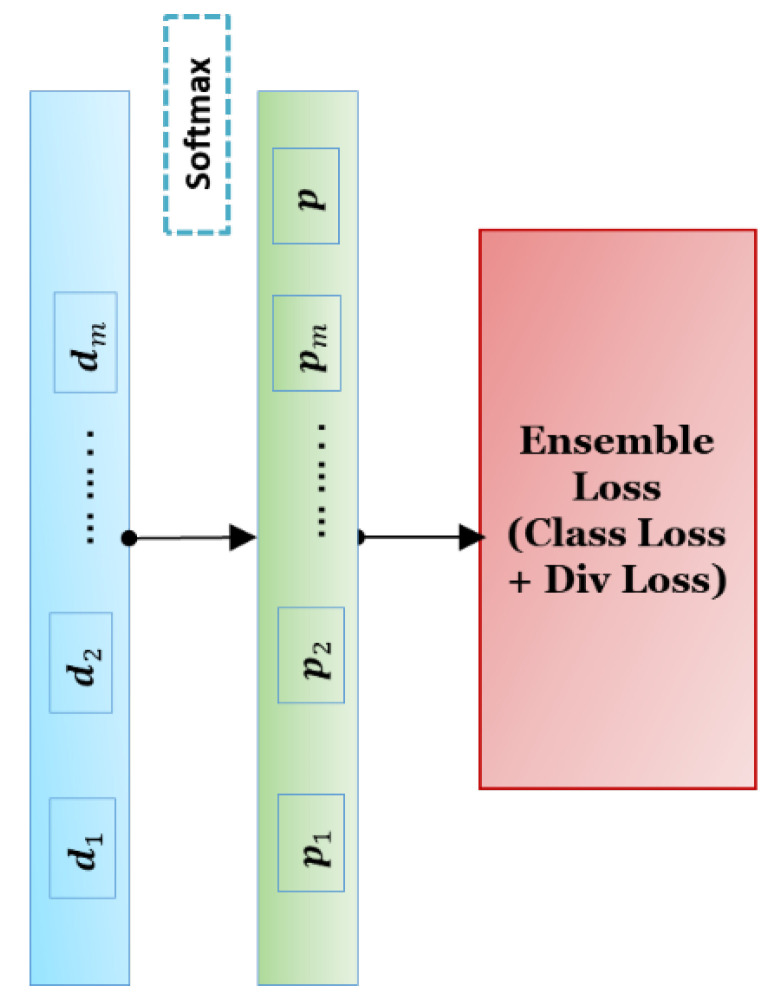
The proposed classification module.

The classifier gets the extracted features (text + image) and outputs a prediction. It consists of a simple FC (fully connected) layer and the proposed loss layer. The loss function plays a key role in the success of our ensemble model. On one hand, it should reduce the classification error on training data, and on the other hand, it should force learners to focus on distinct parts of the text. To this end, we propose the following hybrid loss function:

(7) $$L\left(y_{pred},\;y\right)=\mu L_{class}\left(y_{pred},\;y\;\right)+\left(1-\mu \right)L_{div}\left(y_{pred}\right),$$

where $y_{pred}$ is the output of the model for the input post, and y is a binary variable that shows the true label of the post (0:real, 1:fake). Also $y_{pred}=\left[p_{1},\;p_{2},\ldots ,p_{m},\;p_{m+1}=p\right]$ as seen in Figure 4.

The $L_{class}$ term of loss function should force each learner to have good accuracy. For this purpose, we implement $L_{class}$ as the mean of classification losses of all learners. Another important decision is to select an appropriate classification loss.

Existing methods simply opt for the standard and popular binary cross-entropy (BCE) loss. However, in a real application, the distribution of classes is expected to be imbalanced. For example, two selected real datasets used in our experiment contain more real posts than fake. Thus, in addition to cross-entropy, we examine another loss called focal loss to deal with the imbalanced nature of the target task.

Let $p_{j}^{y}$ be the probability of true class predicted by learner j:

$$p_{j}^{y}=\left\{\begin{array}{c} p_{j},\;\;y=1\\ 1-p_{j},\;\;\mathrm{otherwise} \end{array}\right..$$

The BCE loss between a true label y and prediction of learner j is defined as:

(8) $$\mathrm{BCE}\left(y,p_{j}\right)=-\log p_{j}^{y}.$$

The focal-loss [35] that is more suitable for imbalanced datasets is defined as follows:

(9) $$\mathrm{focal}-\mathrm{loss}\left(y,p_{j}\right)=-{\left(1-p_{j}^{y}\right)}^{\gamma }\log p_{j}^{y}.$$

According to Equation (9), the focal loss adds a factor ${\left(1-p_{j}^{y}\right)}^{\gamma }$ to the cross-entropy loss. Thus, when the hyperparameter is γ>0, it penalizes hard samples $\left(i.e.,\;p_{j}^{y}\ll 1\right)$ more than easy ones. As such, it alleviates the classification bias to the majority classes in imbalanced datasets.

To enforce each learner to achieve good accuracy, we implement the $L_{class}$ as the average of classification losses of all learners:

(10) $$L_{class}\left(y_{pred},\;y\;\right)=\frac{1}{m+1}\overset{m+1}{\underset{i=1}{\sum }}L_{c}\left(p_{i},y\right),$$

where $L_{c}$ is computed using Equation (8) or (9) according to the selected classification loss. 

The $L_{div}$ part of our loss function should enforce the diversity of learners. To this end, we propose the following loss function:

(11) $$L_{div}\left(y_{pred}\right)=\frac{2}{m\left(m-1\right)}\overset{m-1}{\underset{i=1}{\sum }}\overset{m}{\underset{j=i+1}{\sum }}\max\left\{0,\;g+\left(p_{i}-\frac{1}{2}\right)\;\left(p_{j}-\frac{1}{2}\right)\right\},$$

where g is a predefined margin. Note that $\left(p_{i}-\frac{1}{2}\right)\left(p_{j}-\frac{1}{2}\right)<0$ means that predictions are different. When $g+\left(p_{i}-\frac{1}{2}\right)\left(p_{j}-\frac{1}{2}\right)<0$, the loss is zero. Also, when both predictions $p_{i}$ and $p_{j}$e$\left(p_{i}-\frac{1}{2}\right)\left(p_{j}-\frac{1}{2}\right)>0$ and the loss will be increased. Note that the coefficient of the first term should be chosen much larger than the second in practice (μ>0.9). Thus, their combination yields accurate learners with diverse predictions.

### 3.4. Combining the Outputs of Learners

The outputs of m+1 classifiers are passed to the proposed loss function and the computed loss is backpropagated to the layers to train the model. In the evaluation phase, we use the weighted majority voting mechanism to combine the predictions and output the final decision.

To compute the weight of each learner, we keep 20% of the whole training set as validation data. Then, the score of each learner on the validation set is computed. The score function is simply the mean of accuracy and F1-score that makes it suitable for imbalanced datasets. Let $s_{j}$ be the score of learner j. The weight $w_{j}$ is calculated as:

(12) $$w_{j}=\frac{s_{j}}{\sum_{k=1}^{m+1}s_{k}}$$

Finally, the prediction of the given post is computed as:

(13) $$p=Pr\left(y=1\right)=\overset{m+1}{\underset{k=1}{\sum }}w_{j}p_{j}$$

### 3.5. Implementation Details

We implement our model using the *Pytorch* deep learning library. The input news is first cleaned from the redundant characters and split into sentences using the *BeautifulSoup* Python package. Subsequently, the words in the post are tokenized and then converted to a 3D integer tensor as follows:

$$X\in {\mathbb{N}}^{n\times l_{s}\times l_{w}}\;n:\;\mathrm{number}\;\mathrm{of}\;\mathrm{posts}\;\mathrm{in}\;\mathrm{the}\;\mathrm{dataset},\;l_{s}:\mathrm{average}\;\mathrm{number}\;\mathrm{of}\;\mathrm{sentences}\;\mathrm{per}\;\mathrm{post}\;l_{w}:\mathrm{average}\;\mathrm{number}\;\mathrm{of}\;\mathrm{words}\;\mathrm{per}\;\mathrm{sentence}.$$

Here, we consider only the first 50 sentences per post. We use the state-of-the-art XLNet [12] transformer to obtain the embedding for each sentence in the post. XLNet is a breakthrough in NLP as it surpasses the BERT transformer in many downstream NLP tasks. Table 1 shows the specification and architecture of the proposed deep model.

The visual feature extractor includes most parameters in our method (10,295,500/10,940,906 ≈ 94%). Compare to state-of-the-art models used in our experiments: SpotFakePlus [36] and FakeBERT [20], DFDD contains fewer parameters than both SpotFakePlus (with 49,312,552 parameters) and FakeBERT (with 25,555,318 parameters).

## 4. Results and Discussion

This section deals with the experiments conducted to evaluate the effectiveness of the proposed deep model.

### 4.1. Datasets

We adopt the comprehensive fake-news detection repository named FakeNewsNet dataset [36]. The repository is collected from two known sources: Gossip and PolitiFact. Both datasets include news content, image, user comments, and labels obtained by fact checking. We adopt the split test/train in [37] in the experiments (Downloaded from https://drive.google.com/file/d/1Cdil6K5MSU4ebOVrU-4_4DteW8ANNoa2, accessed on 1 February 2022). In the preprocessing step, logos from news were dropped and samples without images or containing GIFs were removed. The statistics of these datasets are summarized in Table 2.

### 4.2. Evaluation Metrics

We utilized standard measures to evaluate the performance of fake-news models including *Accuracy*, *Recall*, *Precision*, *Specificity*, *F1* score, and *G-mean*, defined as follows:

(14) $$Accuracy=\frac{TP}{TP+FP+TN+FN},$$

(15) $$Recall\;\left(Sensitivity\right)=\frac{TP}{TP+FN},$$

(16) $$Precision=\frac{TP}{TP+FP},$$

(17) $$Specificity=\frac{TN}{TN+FP},$$

(18) $$F_{1}=\left(\frac{2}{recall^{-1}+precision^{-1}}\right)=2\frac{precision+recall}{precision\times recall},$$

(19) $$G-mean=\sqrt{Recall\times Specificity}.$$

where *TP*, *TN*, *FP*, and *FN* correspond to true positive, true negative, false positive, and false negative, respectively. G-mean is the square root of the product of class-wise accuracies. It is typically used in an imbalanced environment. A large G-mean value indicates that accuracy on both classes is high and balanced.

### 4.3. Experimental Setup

We compared the proposed DEFD with two peer state-of-the-art methods: FakeBert [20] and SpotFake-Plus [36]. We also evaluate the base variant of our model, named DEFD-base, which has only one learner (m=1).

Both the competing methods only utilize news content to identify its fakeness. The FakeBert only uses the text of a news, whereas the SpotFake-Plus is multimodal and considers both text and visual content of news.

We adopt k-fold (k = 5) cross validation to determine the hyperparameters of the methods. More specifically, we chose the learning rate (lr) from the set $\left\{10^{-4},5\times 10^{-4},\;10^{-3}\right\}$, *optimizer* from $\left\{\mathrm{Adam},\mathrm{RmsProp},\;\mathrm{Ada}-\mathrm{Delta}\right\}$. We fixed the margin g=0.01 and select μ from $\left\{0.93,\;0.95,\;0.97,\;0.99\right\}$, and number of learners m from $\left\{3,\;5,\;7,10\right\}$. 

### 4.4. Results and Discussion

The classification results of the learned models are reported in Table 3 and Table 4. Also, the confusion matrices of the results are illustrated in Table 5.

As the results indicate, DEFD surpasses the competing methods by a large margin on both datasets. For example, on the Gossip dataset, the proposed method obtained an F1 of 72.54%, whereas SpotFake-Plus and FakeBert reached to 49.76% and 24.25%, respectively. This confirms the efficacy of the proposed model. The fact that FakeBert (single modal) achieved the worst results reveals the importance of visual information in identifying fake news. Besides, although FakeBert and Spot-Fake-Plus obtained good specificity (*true negative rate*), they had low recall. A low recall value means that they classified many fake posts as real, that is, they failed to do the primary task of a fake-news detection system. It can be explained as these methods have no strategy to deal with the imbalanced nature of the fake-news identification task. On the other hand, DEFD achieved good balanced class-wise accuracy and has an acceptable recall. It is mainly due to utilizing focal loss function and learning diverse learners that boost the accuracy on minority class (i.e., fake). Meanwhile, the results in Table 3 and Table 4 indicate that the difference between the accuracy of DEFD and DEFED-base is negligible, while DEFD outperforms DEFED-base by a large margin in terms of F1 score and G-mean, which reveals that diversity in learners’ predictions helps to achieve high balanced accuracy and confirms the effectiveness of our ensemble mechanism.

#### 4.4.1. Effects of Visual Information

**Figure 5:**
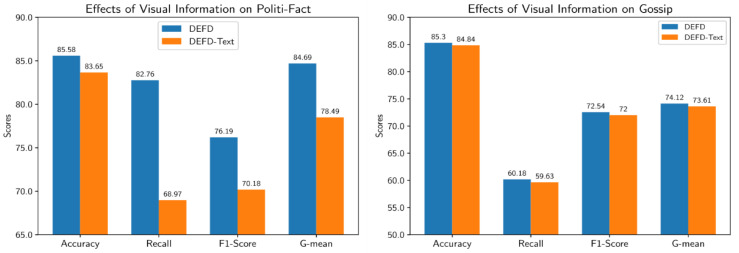
Effects on visual information in fake-news identification on the Politi-Fact and Gossip datasets.

To investigate the role of visual information in the proposed model, we consider a variant of DEFD named *DEFD-Text* that does not include the image feature extractor module. The results obtained by DEFD-Text are compared with DEFD in Figure 5. As seen, in the Politi-Fact dataset, DEFD considerably outperforms DEFD-Text almost in all evaluation metrics. Besides, the improvement obtained by DEFED on the Gossip dataset is not too much. We can conclude that visual information is helpful in identifying fake posts. However, its usefulness highly depends on the dataset.

#### 4.4.2. Hyperparameter Analysis

In these experiments, we analyze the effects of two important hyperparameters in our model: (1) the number of learners (*m*), and (2) the weight of diversity term controlled by the μ. 

**Figure 6:**
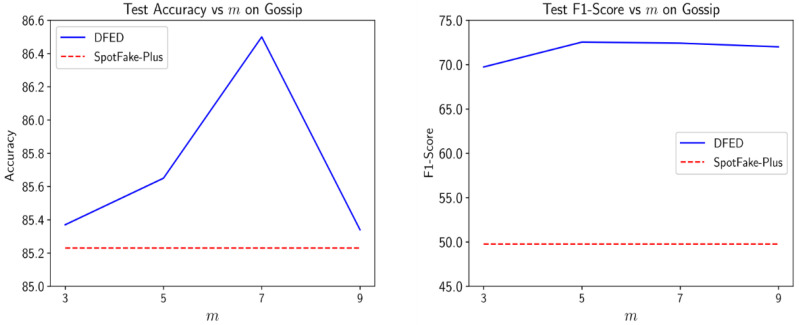
Influence of the number of learners (***m***) in the performance of DEFD on the Gossip dataset.

Figure 6 shows the influence of the number of learners on the accuracy and score of our method on the Gossip dataset. Also, the results were compared with the SpotFake-Plus to provide a better insight into the sensitivity of results to this hyperparameter. As the value of m increases, the complexity of the model grows, and the performance slightly improves. However, setting m greater than the optimal value increases the chance of the overfitting problem and we observe a slight performance reduction. Nevertheless, the obtained results show less sensitivity to the value of m and DEFD outperforms SpotFake-Plus over different values of m.

In the next experiment, we analyzed the effects of the proposed diversity term. Since the value of $L_{div}\;\gg \;L_{class},$ we find out that an appropriate value of μ is near 1. Therefore, we examine the sensitivity of the performance of DEFD to μ by varying it over the set $\left\{0.93,\;0.95,\;0.97,\;0.99\right\}$.

**Figure 7:**
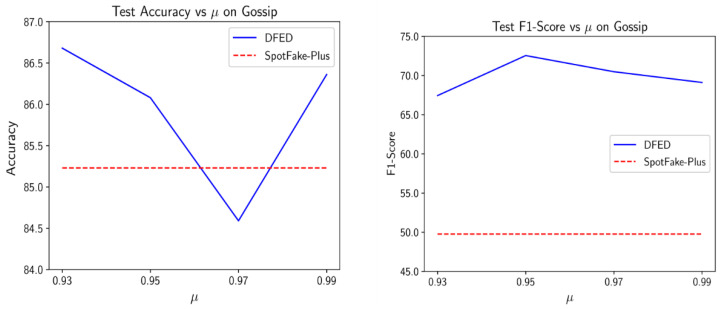
Influence of the weight of the proposed diversity term in the performance of DEFD on the Gossip dataset.

Figure 7 depicts the obtained results versus different values of μ on the Gossip dataset.

As the results indicate, the performance of DEFD peaked around the 0.95 value. However, the difference between obtained scores is not significant, and setting the μ around 1 always yields a satisfactory performance. That is especially obvious when looking at the F1-score measure that embeds two crucial factors in our task (i.e., recall and precision).

## 5. Conclusions

In this research, we studied the multimodal fake-news detection problem solely based on news content, which is considered the best way of early detection of fake posts on social media. Our ensemble model called *DEFD* boosts the performance of existing ones by exploiting both textual and visual cues of the news. DEFD trains diverse learners attending to different aspects of a given post in order to make predictions about its fakeness.

The proposed hybrid loss function forces each learner to have diverse predictions on the one hand and achieve good classification accuracy on the other hand. We also considered the imbalanced nature of fake-news identification tasks and utilized the focal loss function to prevent classification bias to the majority class (i.e., real category).

We simplified the model by building the learners on a common deep-feature extractor, where each learner has only a specific attention module. This architecture reduces the complexity of our model in terms of space and training time requirements and effectively prevents the overfitting problem. Compared to similar models [8,11], we utilized pretrained models, such as XLNET, to directly extract features from the sentences of the input post, which accelerates the training process and decreases the model’s parameters.

Experiments conducted on two real datasets collected from the popular FakeNewsNet repository reveal that the proposed model is indeed effective and surpasses the peer methods by a large margin on both datasets, especially when in addition to accuracy, other classification metrics, such as Recall, F1-score, and G-mean are considered. Also, we analyzed the effect of multiple learners and visual information on the performance of the model through ablation studies. The results confirm that both boost the overall performance of the model. In future work, we aim to extend our work to a cross-dataset scenario. Moreover, extending the work for semisupervised learning (SSL) due to the significant cost of labeling news is valuable. We will also investigate the effects of other attention mechanisms on the model’s performance.

## Figures and Tables

**Table 1:** Specification and architecture of the proposed model (***m*** = **3**) (# mean the number of).

Layer	Input	Output	Connected to	#Parameters
Text Encoder				
Bi-GRU	(50,768)	(50,200)	Inupt Text	522,000
Att-layer1	(50,200)	200	Bi-GRU	40,400
Att-layer2	(50,200)	200	Bi-GRU	40,400
Att-layer2	(50,200)	200	Bi-GRU	40,400
Concat1	(200,200,200)	600	Att-layer1,2,3	-
Image Encoder				
FC ^1^ 1 + Relu	4096	2000	Input Image	8,194,000
FC2 + Relu	2000	1000	FC1	2,001,000
FC3 + Relu	1000	100	FC2	100,100
BN1 ^2^	100	100	FC3	400
Dropout (0.4)	100	100	BN1	-
Classifier				
Softmax1	200	2	Att-layer1	402
Softmax2	200	2	Att-layer2	
Softmax3	200	2	Att-layer3	402
Concat2	(600,100)	600	Att-layer1,2,3	-
Softmax4	700	2	Concat2	1402
				Sum: 10,940,906

^1^ FC: Fully Connected, ^2^ BN: Batch Normalization.

**Table 2:** Specifications of the two real datasets used in our experiments (# mean the number of).

Dataset	#Real Train/Test	#Fake Train/Test	$l_{s}{\;}^{1}$	$l_{w}{\;}^{2}$
Politifact	246/75	135/29	88.18	105.56
Gossip	7974/2036	2285/545	26.28	128.57

^1^$l_{s}$: mean number of sentences per news. ^2^$l_{w}:$ mean number of words per sentence.

**Table 3:** Classification results on the Politi-Fact dataset (the bolde numbers are the maximum value in each column).

Method	Accuracy	Recall	Precision	Specificity	F1-Score	G-Mean
**DEFD**	**85.58**	**82.76**	**70.59**	86.67	**76.19**	**84.69**
**DEFD-base**	83.72	82.61	65.52	84.13	73.08	83.36
**SpotFake-Plus**	78.85	48.28	66.67	**90.67**	56.00	66.16
**Fake-Bert**	77.88	48.28	63.64	89.33	54.90	65.67

**Table 4:** Classification Results on the Gossip dataset (the bolde numbers are the maximum value in each column).

Method	Accuracy	Recall	Precision	Specificity	F1-Score	G-Mean
**DEFD**	85.30	**60.18**	91.29	91.29	**72.54**	**74.12**
**DEFD-base**	**85.43**	52.37	66.19	93.48	58.47	69.97
**SpotFake-Plus**	85.23	33.39	**97.59**	**97.59**	49.76	57.09
**Fake-Bert**	82.12	14.86	65.85	98.16	24.25	38.20

**Table 5:** Confusion matrices of classification results on Politi-Fact (First Row) and Gossip (second row).

DEFD	Predicted Real	Predicted Fake	SpotFake-Plus	Predicted Real	Predicted Fake	FakeBert	Predicted Real	Predicted Fake
Actual Real	65	10	Actual Real	68	7	Actual Real	67	8
Actual Fake	5	24	Actual Fake	15	14	Actual Fake	15	14
**DEFD**	**Predicted Real**	**Predicted Fake**	**SpotFake-Plus**	**Predicted Real**	**Predicted Fake**	**FakeBert**	**Predicted Real**	**Predicted Fake**
Actual Real	2086	199	Actual Real	2230	55	Actual Real	2243	42
Actual Fake	217	328	Actual Fake	363	182	Actual Fake	464	81

## Data Availability

Datasets used in the experiments are publicly available and can be downloaded from the following links: Politi-Fact, Gossip: https://github.com/KaiDMML/FakeNewsNet, accessed on 1 February 2022.
